# Posture-invariant myoelectric control with self-calibrating random forests

**DOI:** 10.3389/fnbot.2024.1462023

**Published:** 2024-12-04

**Authors:** Xinyu Jiang, Chenfei Ma, Kianoush Nazarpour

**Affiliations:** School of Informatics, The University of Edinburgh, Edinburgh, United Kingdom

**Keywords:** EMG, myoelectric control, arm position, transfer learning, self-calibration

## Abstract

**Introduction:**

Myoelectric control systems translate different patterns of electromyographic (EMG) signals into the control commands of diverse human-machine interfaces via hand gesture recognition, enabling intuitive control of prosthesis and immersive interactions in the metaverse. The effect of arm position is a confounding factor leading to the variability of EMG characteristics. Developing a model with its characteristics and performance invariant across postures, could largely promote the translation of myoelectric control into real world practice.

**Methods:**

Here we propose a self-calibrating random forest (RF) model which can (1) be pre-trained on data from many users, then one-shot calibrated on a new user and (2) self-calibrate in an unsupervised and autonomous way to adapt to varying arm positions.

**Results:**

Analyses on data from 86 participants (66 for pre-training and 20 in real-time evaluation experiments) demonstrate the high generalisability of the proposed RF architecture to varying arm positions.

**Discussion:**

Our work promotes the use of simple, explainable, efficient and parallelisable model for posture-invariant myoelectric control.

## 1 Introduction

Myoelectric control has emerged as a promising approach in human-machine interfaces. By recognizing different muscle activities (e.g., hand gestures) via electromyographic (EMG) signals, users can intuitively control exoskeletons or prostheses (Sun et al., 2024; Höhler et al., 2023; Simon et al., 2022; Mereu et al., 2023; Zhang et al., 2023; Leone et al., 2023), and achieve immersive interactions in the metaverse (Lyu, 2023). However, EMG signals show large variabilities due to various confounding factors such as behavior variations, electrode drifting, noises and arm positions. The effect of arm position has received less attentions in previous studies, but can affect real-life applicability of myoelectric interfaces profoundly. In particular, the effect of arm position has been demonstrated to substantially change EMG characteristics (Jiang et al., 2013; Stuttaford et al., 2024) in the aspects of both separability and repeatability (Radmand et al., 2014a), and significantly reduce the classification accuracy by up to ~40% (Fougner et al., 2011).

One approach that previous studies applied to mitigate the arm position effect is involving data collected at multiple arm positions in the decoder training, such that a generalised model can be achieved. This has been via pooling training data from different positions together (Fougner et al., 2011), or developing multiple models at different positions and integrating the parameters of different models (Yu et al., 2017). This category of approaches largely increases the burden of data collection. Other studies aimed to develop a cascade or hierarchical model to first classify the arm positions then adopted position-specific myoelectric control model (Geng et al., 2012; Fougner et al., 2011), which required additional information modalities for arm position recognition. Radmand et al. (2014b) also demonstrated that unless trained in most of possible positions, the integration of inertial data with EMG would significantly degrade the model performance compared with using EMG alone. Kyranou et al. (2024) applied unimodal EMG signals to first estimate the arm position and then use the estimated arm position as a feature in the following hand gesture decoding stage, omitting additional supplementary modalities.

In another recent study, Stuttaford et al. (2024) took an alternative approach to mitigate the arm position effect by improving the consistency of muscle activities across arm positions through user training. They found delayed feedback training contributed to more consistent muscle activities, and such learned motor capability could generalise to untrained positions. In addition, deep neural networks has shown its impressive generalisability in diverse applications. Mukhopadhyay and Samui (2020) showed that a well-trained user-specific deep network can generalise well to different arm positions. However, deep networks are generally data-hungry, that is, they require a relatively large number of samples from each user for model training. A recent study from Meta also developed a large generic deep network-based myoelectric control model (Ctrl-labs at Reality Labs et al., 2024), with training data from 6,527 participants and a total of 60.2 million parameters. Such a large model can hardly be embedded into low-cost mobile computing devices, e.g., microcontrollers.

In this work, we aim to develop a simple, explainable, robust, parallelisable, and computationally efficient model, which at the same time, can generalise well to arm positions and requires minimal training data from the target user. Our previous study (Jiang et al., 2023) demonstrated the excellent explainability and robustness of random forest (RF)-based model in myoelectric control. We validated its robustness against strong noises and corrupted EMG channels. An RF model is also generally considered robust to small sample sizes (Qi, 2012) and easily parallelised owing to the independence of the decision trees. Furthermore in Jiang et al. (2024a), we demonstrated that an RF model is pre-trainable and computationally efficient. In this work, we first prove the inherent superior generalisability of a basic RF model to various arm positions, compared with the benchmark models. Built on our previous work, we then developed a self-calibrating RF model (Jiang et al., 2024b) which can (1) be pre-trained on data from other users, then one-shot calibrated on a new user, and (2) self-calibrate in an unsupervised way to adapt to varying arm positions. Based on our analyses on data from 86 participants (66 for pre-training and 20 for real-time testing), we for the first time demonstrate the high generalisability of a basic RF model to different arm positions, which is a new property of RF model and can advance our understanding on the advantages of RF model in myoelectric control applications. Importantly, we also for the first time prove that the RF model can self-calibrate and progressively improve its performance even during arm rotation, by learning generalised knowledge from highly dynamic data distributions at varying arm positions.

## 2 Materials

### 2.1 Ethical approval

All participants signed an informed consent form before taking part in the experiment, which was approved by the local ethics committee at the University of Edinburgh (ref: 2019/89177).

### 2.2 Pre-training dataset

**Figure 1:**
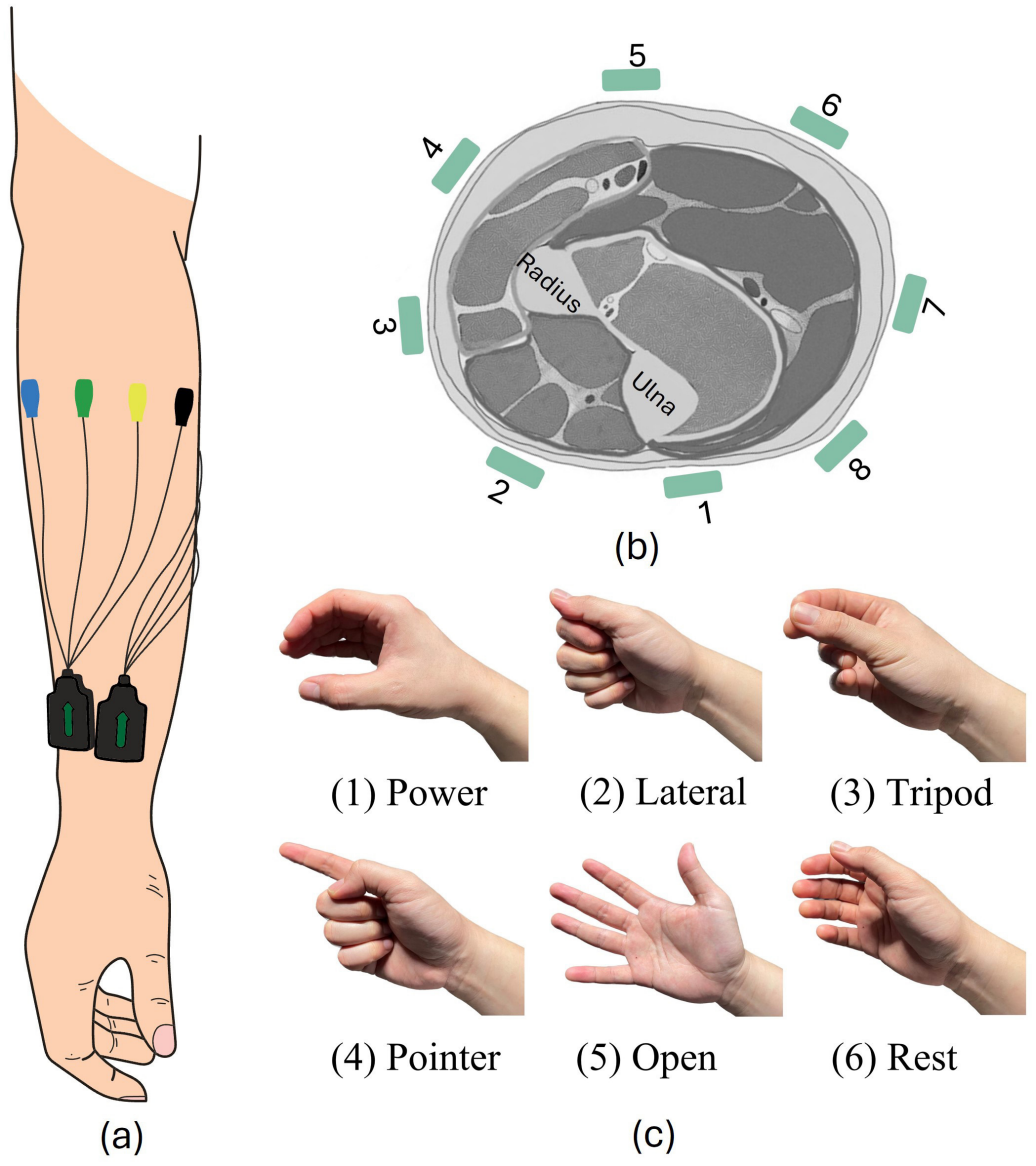
Experiment setup. **(A, B)** EMG electrode positions. **(C)** All six hand gestures involved in our experiment.

We combined data that we had collected in three different experiments. These datasets were collected from 66 participants. For all datasets, EMG data was recorded using eight Trigno™ electrodes (Delsys, Inc., USA), which were placed on participants' right forearm with equal inter-electrode distances, as presented in Figures 1A, B. EMG data was collected during six hand gestures, as shown in Figure 1C.

*Dataset 1* comprised data from 20 participants (22–43 years old, 12 males, 8 females). Each participant performed 10 trials for each hand gesture (4s valid duration each trial), with a 5s inter-trial resting period.

*Dataset 2* comprised data from 18 *new* participants (22–28 years old, 11 males, 7 females). For each participant in dataset 2, EMG signals with 51s duration were recorded for each hand gesture in 51 trials (1s signal duration each trial).

*Dataset 3* was collected from 28 *new* participants (21–42 years old, 13 males, 15 females) following a similar data collection paradigm as dataset 2. For each participant in dataset 3, EMG signals with 51s duration were recorded for each hand gesture in 51 trials (1s signal duration each trial).

Detailed descriptions of datasets can be found in Jiang et al. (2024a,b). All pre-training data were collected with participants' arm pointing vertically toward the ground, in an open-loop setting.

### 2.3 Real-time myoelectric control experiment

The real-time myoelectric control experiment was designed to test the performance of pre-trained and self-calibrating RF model.

We recruited 20 *new* participants (19–31 years old, 10 males, 10 females). The experiment for each participant consists of a calibration session and a testing session. The calibration session consists of only one 2s trial for each hand gesture. A 2s inter-trial resting period was provided. The setting with only one calibration trial per hand gesture was designed to validate the performance of one-shot model training/calibration. In each 2s trial, participants could react to the visual cue and shape their hand in one second, and then hold the target hand gesture in the next second. Only EMG signals recorded during the 1s gesture holding period were used in following analyses (the same for the following parts of the real-time experiment with a 2s trial duration). The collected calibration data were used to calibrate pre-trained models. Then the calibrated models were implemented in the following testing session, outputting and saving the hand gesture decoding outcome in real-time (every 100 ms). The experiment was conducted in an open-loop mode, in which the classification outcomes were not presented to participants, so that our experiment did not induce any motor learning. In this way, different models could be implemented simultaneously and compared. Two models (with and without self-calibration) were implemented. Details of self-calibration will be introduced in the following section. The testing session consists of 11 testing blocks, with 5 trials (2s trial duration) per hand gesture (30 trials for all hand gestures) in each testing block. The order of 30 trials for all hand gestures within a testing block was generated randomly. Participants were provided with a 2s inter-trial and 5 minutes inter-block resting period.

**Figure 2:**
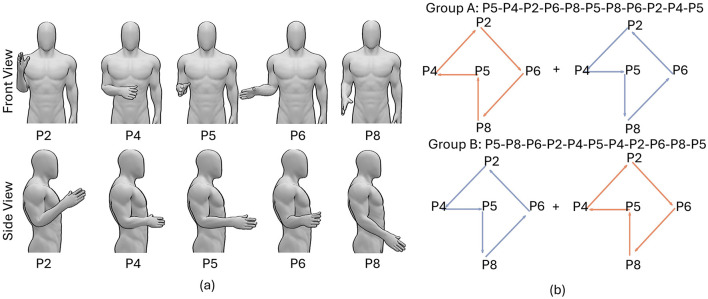
The paradigm of the real-time experiment during which we tested the efficacy of a self-calibrating RF model to account for EMG distribution shift because of varying arm positions. **(A)** Postures with different arm positions. Both front and side views are presented. **(B)** The sequences of arm positions in blocks 1–11 in group A and group B. Red and blue colors correspond to rotating arm clockwise and counterclockwise, respectively.

Data in the calibration session was collected with participants' elbow positioned at a angle of 90 degrees, defined as the position 5 (P5) in Figure 2A. The arm positions in the testing sessions were fixed in a single block but varied in different blocks.

The 20 participants were separated into two groups: group A and group B, with participants in group A rotating their arm first clockwise and then counterclockwise while participants in group B rotating their arm first counterclockwise and then clockwise, as presented in Figure 2B. The postures with different arm positions are presented in Figure 2A. Such experiment design generates a symmetrical arm position sequence for each group. For example, the arm position sequence for group A in 11 testing blocks was: P5-P4-P2-P6-P8-P5-P8-P6-P2-P4-P5, symmetrical around the middle block. The symmetrical sequence in each group and the combination of two sequences in both groups, can to the large extent remove the time factors when comparing the average decoding accuracy at different arm positions.

## 3 Methods

### 3.1 Feature extraction

Features were extracted from EMG signals in each channel via a 200ms sliding window at a 100 ms sliding step. In each window and each channel, first, mean absolute value (MAV), waveform length (WL), root mean square (RMS), slope sign changes (SSC), and zero crossings (ZC) were extracted as energy features (Englehart and Hudgins, 2003). These energy features are the most commonly used ones in previous studies and their effectiveness has been validated in diverse myoelectric control applications (Englehart and Hudgins, 2003; Ye et al., 2021; Hudgins et al., 1993). Second, the skewness was extracted as a distribution feature (Nazarpour et al., 2007). Third, the peak frequency (PKF), median frequency (MDF), mean frequency (MNF), and variance of central frequency (VCF) were extracted as spectrum features (Phinyomark et al., 2012). The above energy, distribution, and spectrum descriptors represent the EMG signals from different aspects and their complementary roles have been validated in previous work (Jiang et al., 2024a). All features form a 80-length feature vector for the 8-channel EMG within each window.

### 3.2 RF model pre-training and fine-tuning

Data from 66 participants were used to pre-train a RF model. EMG features were first normalised separately for different participants via z-score. To encourage diversity among decision trees and at the same time constraining the number of nodes, only 2% samples were randomly picked via bootstrapping to build each decision tree. The pre-trained RF model comprised 200 decision trees.

We further performed 2 operations to fine-tune the pre-trained RF model, namely decision tree pruning and decision tree appending. To prune a pre-trained decision tree, the calibration data collected from a new target participant were used as a validation dataset to remove unnecessary and inaccurate nodes. For each decision tree, the pruning operation was first performed on the leaf nodes with the longest distance away from the root node. A node together with its children nodes would be removed if the validation performance without these nodes would not degrade. We then inspected all nodes upwards until the root node was also inspected. Details on the decision tree pruning operation were reported in Jiang et al. (2024a).

In addition, we appended another 200 participant-specific decision trees trained from scratch using only the calibration data (1 repetition per hand gesture) from the target participant. The 200 pre-trained and pruned decision trees and 200 appended decision trees together form a fine-tuned RF model with 400 decision trees.

### 3.3 RF model self-calibration

**Figure 3:**
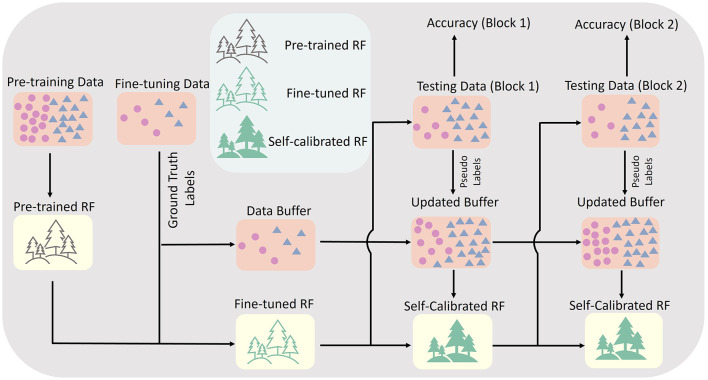
The framework of self-calibrating RF common model.

With the pre-trained and fine-tuned RF model, we enabled an unsupervised self-calibration for the RF model after each testing block. This features augmented the RF model to adapt to the varying data distribution due to the arm position effects autonomously. The core framework of our self-calibration approach is presented in Figure 3. During each testing block, a data buffer was used to store testing data. The data buffer could store up to 1,500 testing samples (features extracted in each sliding window), equivalent to ~500 KB storage size if using 32-bit floating point data format. If the number of stored testing samples reached the upper bound of the buffer, the oldest stored sample belonging to the class category with the most samples were removed from the buffer, and replaced by the latest one. After each testing block, all data in the data buffer were first mapped into a 3-dimensional subspace with a more simple distribution via manifold learning using t-Distributed Stochastic Neighbor Embedding (t-SNE) (Maaten and Hinton, 2008). Then, the K-Means method was applied to assign pseudo-labels to all stored samples. The initialisation of K-Means labels was set as the predicted labels of the current model. The final number of samples in the data buffer with different hand gesture pseudo-labels might be different. To built a balanced self-calibration dataset, the number of latest samples for each hand gesture used in the following step was set as the lowest number of samples for each hand gesture. The stored testing samples with pseudo-labels and the calibration samples with ground-truth labels (already used in the fine-tuning stage) were mixed together as a self-calibration dataset. Then, 40% of the appended decision trees were randomly selected, and replaced by new appended decision trees trained using the latest self-calibration dataset. The self-calibrated RF model, together with the fixed fine-tuned RF model, were implemented in the next testing block.

### 3.4 Ablation experiment

To evaluate the contributions of different components in the framework of our method, the ablation experiment was performed by quantifying the reduction in accuracy with each component removed from the framework. The ablation experiment was run offline by replaying exactly the same testing data collected from 20 participants in our real-time experiment. These components comprise: the energy features, the distribution features, the spectrum features, the K-Means clustering, and the t-SNE manifold learning. Note that when removing the t-SNE manifold learning, the pseudo-labels were assigned by performing K-Means on the original high-dimensional feature space. When removing K-Means, the pseudo-labels were assigned simply as the output labels of the current model (t-SNE was also removed in this case as it was not necessary without the following clustering-based pseudo-label assignment).

### 3.5 Baseline models

First, the standard participant-specific RF model (400 decision trees) trained using only the calibration data (one repetition per hand gesture) from the target participant was implemented offline. In addition, as one important purpose of our work is to provide a computationally efficient alternative for myoelectric control applications, the participant-specific linear discriminant analysis (LDA) and support vector machine (SVM) models, were implemented offline as efficient benchmark models, due to their effectiveness and computational efficiency on mobile computing devices.

### 3.6 Validation methods

Pre-training data from the 66 participants were used offline to pre-train a RF model. Data from the calibration session of the target testing participant in our real-time experiment were used to fine-tune the pre-trained RF model, or train a standard RF, LDA, and SVM baseline models from scratch. All RF models in our work consist of 400 decision trees as further increasing the number of decision trees would not contribute to a substantially improved accuracy but increase the model complexity. For SVM, linear kernel was used due to its simplicity and better classification performance. All hyper-parameters were determined using data from pre-training dataset (66 participants) and directly applied on data from the 20 testing participants, so that the obtained model performance was not over-estimated. Note that the pre-trained and fine-tuned RF model and the self-calibrating RF model were implemented in real time, while the performances of baseline models were evaluated offline on the same testing data collected in real-time experiment. All accuracies were calculated by comparing the model-predicted label and the ground-truth label on EMG within each sliding window during the gesture holding period (the last 1 s period of a trial).

### 3.7 Statistical analyses

Considering multiple arm positions were involved in our evaluation, the Friedman test was first applied to verify the global significance among all groups. Then the Nemenyi test, a *post-hoc* test was applied to find the pairwise significance of each two groups. Significance was claimed for *p* < 0.05.

## 4 Results

### 4.1 The effect of arm position on EMG characteristics

**Figure 4:**
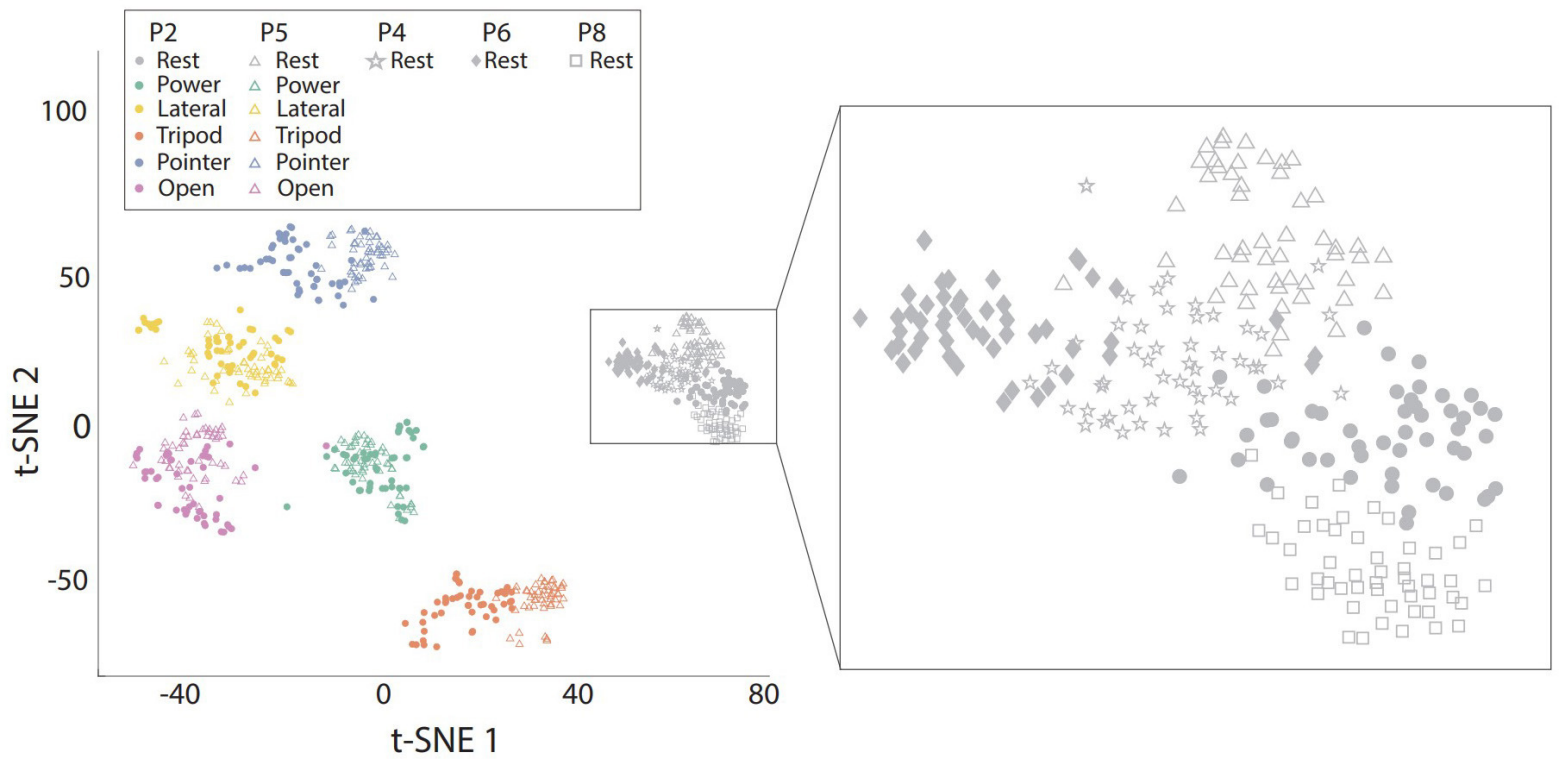
The visualisation of EMG feature distribution from one representative participant in a 2-dimensional space obtained by t-SNE. **Left**: Data from all hand gestures at two positions, namely P2 and P5; **Right**: EMG feature distribution of the Rest class at all five arm positions.

To better and intuitively illustrate the effect of arm position on EMG characteristics, we first visualise the distribution of EMG features in a 2-dimensional space obtained by t-SNE, as presented in Figure 4. The arm position effect leads to the shift of distribution for the same hand gesture, particularly for tripod, pointer and rest hand gestures. We further zoomed on the distribution of the same hand gesture (rest) at all 5 arm positions. According to the right panel of Figure 4, the arm position effect leads to variability of baseline muscle activity of the “rest” hand gesture.

**Figure 5:**
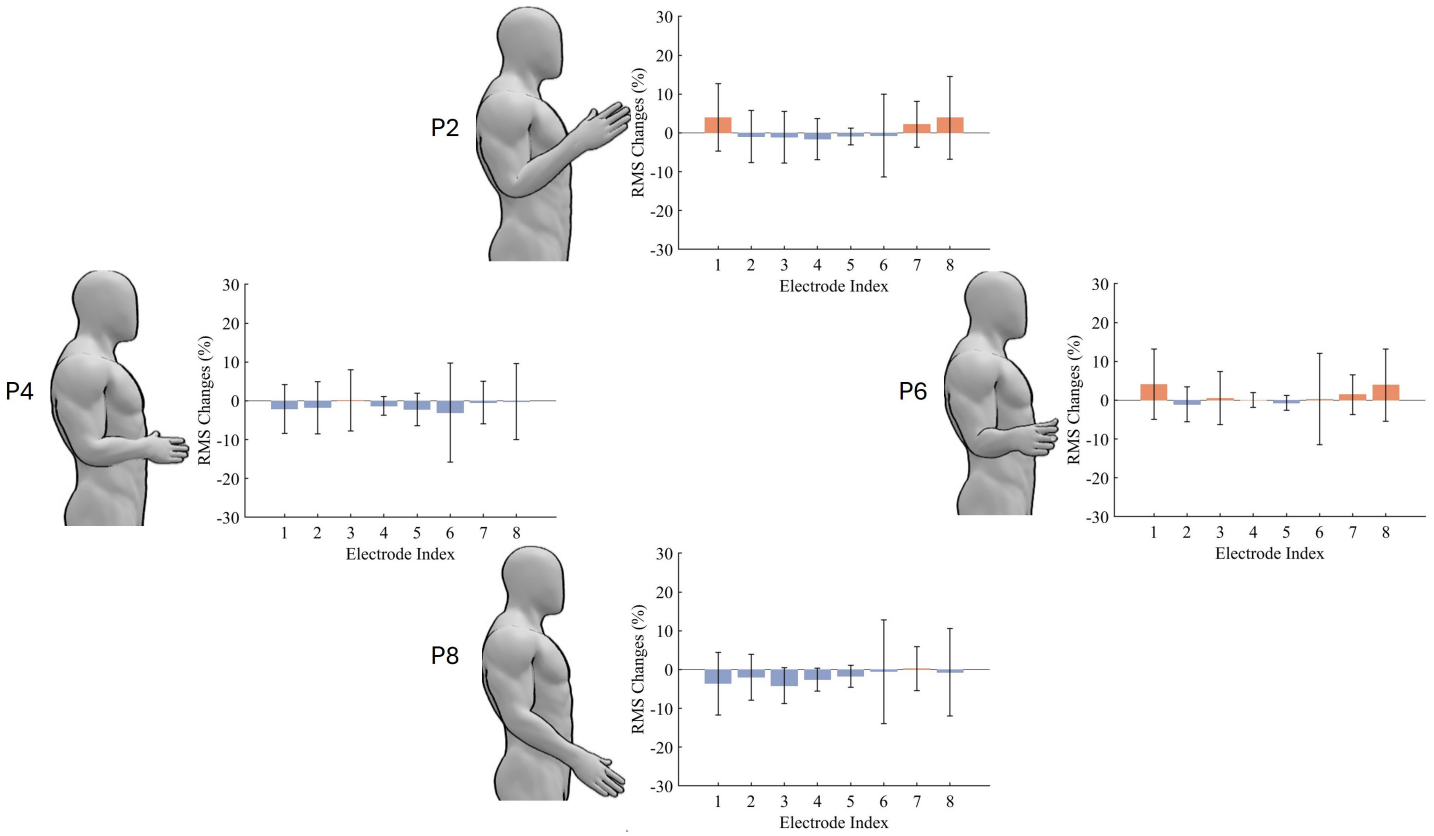
The Effect of arm position on muscle activity. The presented RMS values are changes relative to P5. Red and blue colors represent increased and decreased muscle activities, respectively.

With the intuitive visualisation of the arm position effect on the overall feature distribution, we wondered if we can see the change across a specific feature as well at the electrodes. Figure 5 presents the variability of muscle activity (represented by RMS) with varying arm positions. Overall, arm positions P2 and P6 contribute to relatively more muscle activities, while P4 and P8 help participants save muscle efforts. In addition to average muscle activities, the variation level of muscle activity changes measured by the standard deviation (STD) also vary with different electrodes and arm positions. Specifically, electrodes 4 and 5 contribute to a lower STD but electrodes 6 and 8 contribute to a higher STD.

### 4.2 The robustness of different models against arm positions

**Figure 6:**
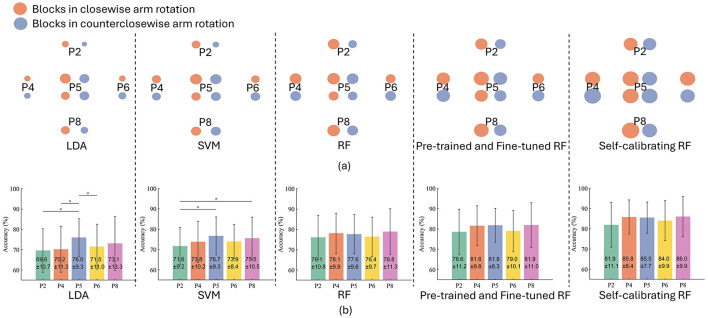
Classification results of different models. **(A)** Intuitive visualisation of performances of different models. The circle size refers to the accuracy (linearly correlated). The average accuracy in the clockwise and counterclockwise conditions are presented separately. **(B)** The classification accuracy of different models, where “*” denotes a significant difference.

Figures 6A, B present intuitive visualisation of and quantitative comparison between the classification performances of different models, respectively. By comparing the classification performances between LDA, SVM, and standard RF in Figure 6B, we can find that all models contribute to a similar level of accuracy when trained and tested at the same arm position (P5), with an average accuracy of 76.0 ± 9.3%, 76.7% ± 9.3%, and 77.6 ± 9.6% achieved by LDA, SVM, and RF, respectively. However, if tested at a different arm positions (other than P5), the classification accuracy of LDA would substantially reduced, by 6.4% at P2, 5.8% at P4, 4.5% at P6, and 2.9% at P8, with a statistical significance observed at P2, P4, and P6. Likewise, the accuracy of SVM would decreased by 5.1% at P2, 2.9% at P4, 2.8% at P6, and 1.2% at P8, with the accuracy at P2 significantly lower than P5 and P8. However, standard RF shows a small reduction in classification accuracy (<1.5%) when tested at a arm position different with that during model training. Overall, the standard RF model does not result in significantly different accuracy at different arm positions, demonstrating its inherent generalisability and robustness to the arm position effect.

With the inherent robustness to arm position effect, the additional pre-training and then fine-tuning process can improve the accuracy by 2.5% at P2, 3.5% at P4, 4.2% at P5, 2.6% at P6, and 3.1% at P8. Moreover, the self-calibration module can further improve the classification accuracy by 3.3% at P2, 4.2% at P4, 3.7% at P5, 5.0% at P6, and 4.1% at P8. The progressively improved performance of RF-based models with pre-training, fine-tuning and self-calibration demonstrates the effectiveness of the whole framework of our self-calibrating RF common model.

### 4.3 Effects of the accuracy of pseudo-labels

**Figure 7:**
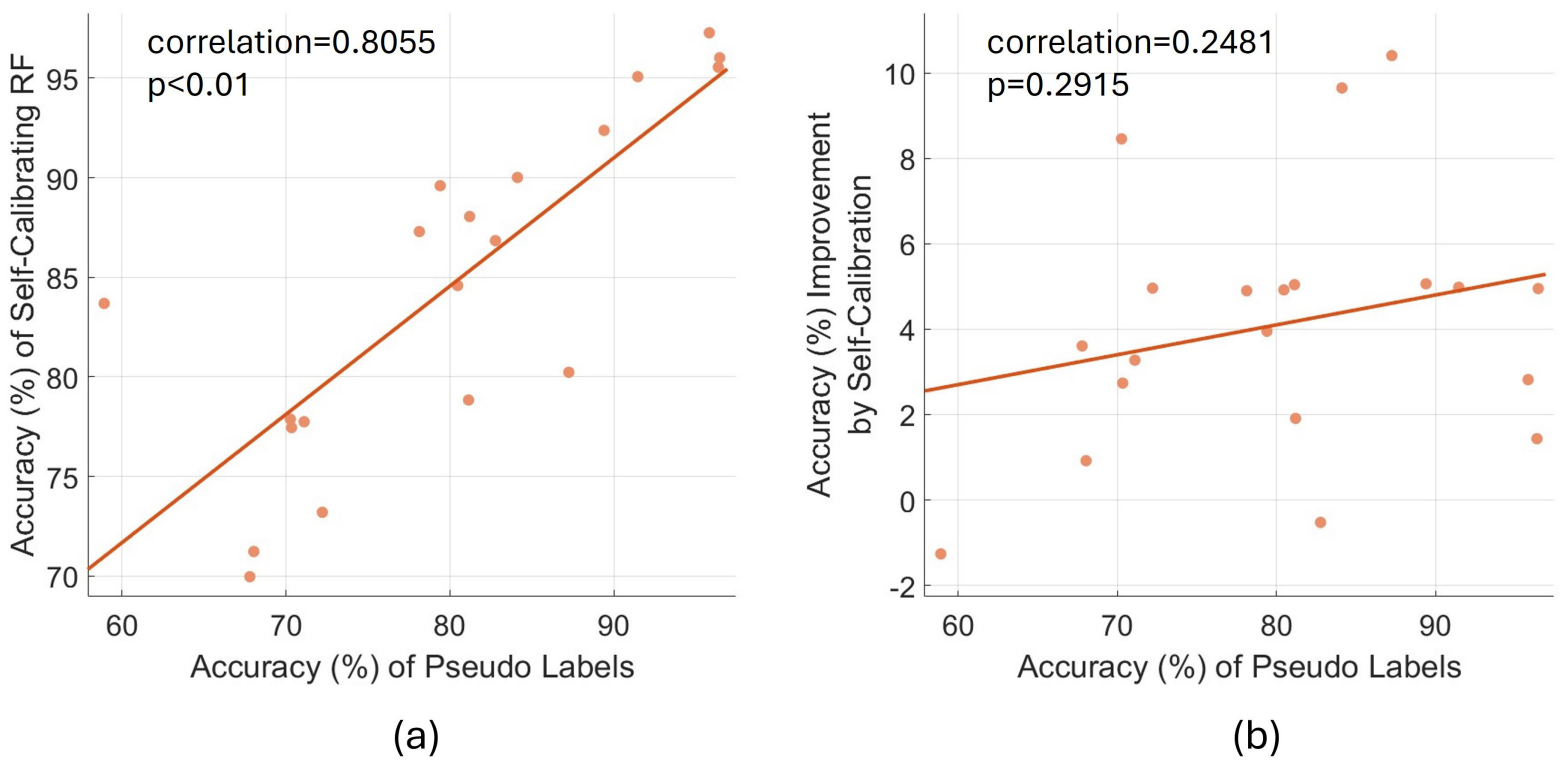
The relation between the accuracy of pseudo-labels, the accuracy of self-calibrating RF and the accuracy improvement by self-calibration. **(A)** The relation between the accuracy of pseudo-labels and the accuracy of self-calibrating RF. **(B)** The relation between the accuracy of pseudo-labels and the accuracy improvement by self-calibration (i.e., the accuracy of self-calibrating RF minus the accuracy of pre-trained and fine-tuned RF). Each data point refers to the overall average results of a specific subject. The plotted straight line is the linear fit of all data points.

We also evaluated the average accuracy of pseudo-labels of data samples used to self-calibrate the model after each testing block, with an average accuracy of 80.1% achieved. Figure 7 presents the variation of both the accuracy of self-calibrating RF and the accuracy improvement by self-calibration, with varying accuracy of pseudo-labels. According to Figure 7A, the accuracy of pseudo-labels is positively correlated with the accuracy of self-calibrating RF (linear correlation coefficient: 0.8055; *p* < 0.01). To better illustrate the effect of pseudo-label accuracy, we then quantified the accuracy improvement of the self-calibrating model compared with the pre-trained and fine-tuned (fixed) RF model, with varying accuracy of pseudo-labels. According to Figure 7B, generally, more accurate pseudo-labels tend to improve model performance to a higher degree, but without significance (linear correlation coefficient: 0.2481; *p* = 0.2915).

### 4.4 Results of the ablation experiment

To evaluate the contribution of each component in the framework of our method, we performed an ablation experiment with results presented in Table 1. Among all features, removing energy features contributes to the most substantial accuracy reduction (*p* < 0.05), demonstrating the great contribution of energy features in the framework. Removing distribution features or spectrum features also slightly degrade the model performance (without significance), demonstrating that these features would to a certain degree further refine the decision making logic of the model on the basis of energy features. Additionally, assigning pseudo-labels without t-SNE manifold learning or K-Means results in degraded model performance (without significance). Even though the contribution of an individual component might not be obvious, their joint contribution results in a highly effective, robust, and generalisable model.

**Table 1:** Results of the ablation experiment.

**Energy features**	**Distribution features**	**Spectrum features**	**K-Means Clustering**	**t-SNE manifold learning**	**Accuracy (%)**
✗	✓	✓	✓	✓	71.4 ± 8.1
✓	✗	✓	✓	✓	83.9 ± 8.7
✓	✓	✗	✓	✓	84.2 ± 8.6
✓	✓	✓	✗	✗	82.7 ± 9.4
✓	✓	✓	✓	✗	81.0 ± 9.3
✓	✓	✓	✓	✓	**84.6** **±** **8.5**

All results in this table were obtained offline by replaying exactly the same testing data collected from 20 participants in our real-time experiment. Note that when removing K-Means, the pseudo-labels were assigned simply as the output labels of the current model (t-SNE was not applicable in this case so it was removed together with K-Means). The bold value represents the best performance.

## 5 Discussion

In this work, we showed the excellent generalisability of RF models to different arm positions, and demonstrate a substantial accuracy improvement achieved via RF pre-training, fine-tuning, and self-calibration. The effect of arm position is a multi-faceted problem. First, different arm positions contribute to different baseline muscle activity, validated by the feasibility to classify different arm positions using EMG with a relaxed hand gesture (Asghar et al., 2022). Second, in addition to the effect on baseline muscle activity, the user behaviours to perform different hand gestures may vary at different arm positions, supported by the fact that motor learning with delayed feedback to improve the behaviour consistency can mitigate the arm position effect (Stuttaford et al., 2024). The above effects of arm positions may also partly result from changes in load applied to muscle tendons due to gravity (Jiang et al., 2013). Changes in gravity load at different arm positions further lead to different levels of gravity-compensatory muscle activity. Additionally, diverse muscle groups locate at the forearm, the relative positions of which may change considerably during arm movement. The shift of the relative positions of muscle with respect to each EMG electrode may also change with varying arm positions (Jiang et al., 2013). All above factors explain the effect of arm position on EMG characteristics.

The arm position effect is considered as one of the confounding factors that lead to the variability of EMG and its distribution shift. It is not realistic to collect and include EMG data from all possible arm positions in the training dataset. Our work validated the inherent superior generalisability of a basic RF model to various arm positions. Such generalisation might results from the “divide and conquer” training strategy of a RF model. First, each decision tree selects only a small subset of features, and the overall feature distribution can be learned through an ensemble of all decision trees. Additionally, different arm positions contribute to the shift of certain but not all features, evidenced by the nearly zero muscle activity changes at certain channels in Figure 5. Therefore, the variation of a subset of features would only degrade the performance of a small proportion of decision trees, with the overall performance of the RF model kept at a relatively stable level. Furthermore, in the inference process of each decision tree, the value of each feature is used to simply compare with a threshold, without complex calculation to enlarge and propagate the error in the slightly shifted feature. By contrast, for LDA, all model parameters are dependent with each other. The shift on a specific feature due to the arm position effect would be enlarged by the calculation operations in the inference process using all LDA parameters and all EMG features. Moreover, the self-calibrating model proposed in this work can automatically adapt to and, most importantly, learn extra knowledge from new arm positions. Moreover, the initial model parameters can be determined by only one repetition (1s signal duration) at only one arm position (P5) for each hand gesture from each new target user. Together with the work of Stuttaford et al. (2024), it is evident that the combination of an adaptive decoder and user adaptation can mitigate the arm position effect.

Previous studies have proposed diverse transfer learning algorithms to improve the generalisability of myoelectric control models, e.g. by supervised model fine-tuning (Cŏté-Allard et al., 2019) or unsupervised domain adaptation (Liu et al., 2024; Shi et al., 2024). These transfer learning algorithms have been proved effective in mitigating the confounding factors such as the inter-user (Cŏté-Allard et al., 2019) and inter-day EMG variabilities (Shi et al., 2022), electrode shift (Chan et al., 2022) etc. Transfer learning methods have been proved effective in addressing above factors by performing one-time model calibration before each use, because once the model is calibrated, such factors remain relatively stable in each setup. By contrast, the effect of arm position is more dynamic and unstable in practical applications. The arm position may change dramatically within a short period of time. Therefore, one-time model calibration methods cannot adaptively track the shift of data distributions during each use. Accordingly, in previous studies, the most effective way to address such dynamic arm position effect is to develop a model robust to arm positions, using training data collected at most of possible arm positions. In this work, we first prove that, a basic RF model is inherently robust to arm positions even if the model is trained on a small dataset at a fixed arm position. Then, we demonstrate that unsupervised model self-calibration can adaptively track the dynamic data distributions at varying arm positions, and can even improve the model performance automatically during arm rotation.

Previous studies on unsupervised model calibration mainly performed offline validations by pooling all testing data together, instead of considering the confounding factors (e.g., arm position) as a dynamic effect which can change within a short period of time. This is the first study to conduct a real time experiment to validate the performance of our self-calibrating model with dynamic varying arm positions. Both the supervised one-shot model fine-tuning and the unsupervised model self-calibration in our work are implemented on a simple RF model, which is parallelisable and robust to small sample size (Qi, 2012), providing an simple but effective alternative in practice. All these conclusions can advance our understanding on the advantages of a simple RF model in myoelectric control applications.

In practical myoelectric control applications, users need to bear the weight of prosthetic devices. At different arm positions, users need different levels of extra muscle efforts to support the prosthetic devices. Arm position would become a more important confounding factor in prosthetic control applications. In this case, the developed model robust to arm positions would show higher significance. Moreover, prosthetic devices are usually applied in mobile scenarios with constrained computing resources. The developed RF model comprises multiple independent decision trees which are easily parallelisable, and thereby is easily implemented in a computationally efficiency way.

As for the technical details of our self-calibration module, we assign pseudo-labels on testing samples by jointly considering the knowledge learned by the current model (for label initialisation in clustering) and the overall distribution of stored testing samples (via manifold learning and clustering). By contrast, directly assigning pseudo-labels as the output labels of the current model without manifold learning and clustering, may likely lead to a loop of learning biased knowledge given by the model itself. The clustering-based pseudo-label assignment serves as a biased-error correction mechanism in the pseudo-labeling process. As for the one-shot fine-tuning process, the pruning operation performed on pre-trained decision trees could integrate both the generalised information from many users in the database and the specific information from the target user. The decision tree appending operation further augments the contributions of the personalised, but scarce, fine-tuning data from the target user. The supervised one-shot fine-tuning and the unsupervised self-calibration modules progressively improve the model performances.

Note that the proposed model is validated on limb-intact subjects. Performance validations with people with limb difference are required in future studies. We assume that, the EMG characteristics of people with limb difference and limb-intact people share certain similarities. Previous studies (Fan et al., 2023; Lin et al., 2023) have shown that a myoelectric control model can learn useful knowledge from the data of limb-intact subjects, to help improve the model performance on amputees. Accordingly, we expect our proposed method validated on limb-intact subjects would, to some extent, also contribute to more robust prosthetic devices for amputees.

## 6 Conclusion

In this work, we demonstrated the inherent generalisability and robustness of standard RF model to varying arm positions. Moreover, we developed a self-calibrating RF model which can be pre-trained on data from other users, and conveniently personalised on a new user using only one repetition per hand gesture with only 1s of signal. During the testing phase, the model calibrated at a fixed arm position can autonomously track the varying data distribution at different unseen arm positions, and even improve the classification accuracy, unlike the performance degradation that previous static models suffered from. Together with the inherent explainability, parallelisability, computational efficiency, and robustness to noises of RF, we expect our self-calibrating RF model largely benefit the translation of myoelectric control into real world practice.

## Data Availability

The original contributions presented in the study are included in the article/supplementary material, further inquiries can be directed to the corresponding author.
